# Sex differences in patient mortality after traumatic spinal cord injury: A systematic review and meta-analysis

**DOI:** 10.1016/j.bas.2026.106176

**Published:** 2026-07-16

**Authors:** Elise Beijer, Floor J. Mansvelder, Romein W.G. Dujardin, Nicole P. Juffermans, Linda J. Schoonmade, Leo M.G. Geeraedts, Frank W. Bloemers, Patrick Schober, Charissa E. van den Brom

**Affiliations:** a Dept. of Surgery, Section Trauma Surgery, Amsterdam University Medical Centers, VU University Amsterdam, de Boelelaan 1117, Amsterdam, the Netherlands; b Dept. of Anesthesiology, Amsterdam University Medical Centers, VU University Amsterdam, de Boelelaan 1117, Amsterdam, the Netherlands; c Laboratory of Experimental Intensive Care and Anesthesiology (LEICA), Amsterdam University Medical Centers, University of Amsterdam, the Netherlands; d Dept of. Intensive Care Medicine, Erasmus MC, University Medical Center Rotterdam, Dr. Molewaterplein 40, Rotterdam, the Netherlands; e Medical Library, VU University Amsterdam, Amsterdam, the Netherlands; f Helicopter Emergency Medical Service Lifeliner 1, Westelijk Havengebied, Amsterdam, the Netherlands; g Dept. of Intensive Care Medicine, Amsterdam University Medical Centers, University of Amsterdam, Amsterdam, the Netherlands

**Keywords:** Traumatic spinal cord injury (TSCI), Trauma injury, Sex differences, Mortality, Survival

## Abstract

**Introduction:**

Traumatic spinal cord injury (TSCI) causes high mortality and long-term disability, with profound physical, emotional, and socioeconomic impact. While males represent the majority of cases, females may have lower mortality, potentially due to neuroprotection and immune modulation. However, the impact of sex on mortality remains unclear, limiting opportunities for sex-specific management.

**Research question:**

To evaluate the association between sex and mortality after TSCI.

**Material and methods:**

PubMed, Embase, Web of Science, and the Cochrane Library were systematically searched on June 4, 2025. Observational studies reporting sex-stratified mortality in TSCI across all ages and injury severities were included. Screening and data extraction were performed independently by multiple reviewers. Random-effects meta-analysis calculated odds ratios (OR) for mortality in females versus males, with leave-one-out sensitivity analyses. Heterogeneity was assessed using I^2^ and prediction intervals. Study quality was evaluated using an adapted Newcastle-Ottawa Scale. PRISMA and MOOSE guidelines were followed.

**Results:**

Ten studies including 63,992 patients (18,788 females and 45,204 males) were analyzed, with no evidence of small study bias. Sex was not significantly associated with TSCI mortality (OR 0.86, 95% CI 0.69-1.07, p = 0.18). Sensitivity analyses and qualitative evaluation of individual studies (six of ten, adjusted analyses) indicated lower mortality in females. Substantial heterogeneity was observed (I^2^ 79.8%).

**Discussion and conclusion:**

Overall, sex was not associated with TSCI mortality. Nevertheless, individual studies and sensitivity analyses indicate lower mortality in females. These findings appear context-dependent, underscoring the need for future research to clarify when sex influences outcomes, supporting individualized risk stratification and sex-specific management.

## Highlights

• This study found no singular overall association between sex and TSCI mortality
• Sensitivity analyses imply lower female mortality, confirmed in qualitative synthesis
• The association between sex and mortality after TSCI appears context-dependent
• Future research should clarify conditions under which sex influences TSCI outcomes

## 1 Introduction

Traumatic spinal cord injury (TSCI) leads to considerable mortality and morbidity (Lu et al., 2024; Ahuja et al., 2017; Ding et al., 2022) and due to limited treatment options, it results in profound physical, emotional, and economic impacts on patients, their families, and society (James et al., 2019; Tian et al., 2023; van den Berg et al., 2010; Van Den Berg et al., 2011). The reported incidence of TSCI is estimated at 47 per million people (Lu et al., 2024), with approximately 80% of cases occurring in males (Shank et al., 2019; Chen et al., 2016), highlighting a significant sex-related difference in incidence.

Multiple factors influence mortality after TSCI, with higher-level injuries increasing the risk of respiratory complications and poor long-term outcomes. Patient-related factors, including sex, may also affect survival, as females and males may differ in physiological response to spinal cord injury, including neuroinflammatory responses, nerve injury patterns, and locomotor recovery (Stewart et al., 2021; Li et al., 2022; Osimanjiang et al., 2022; Datto et al., 2015; Benedict et al., 2026). Clinical evidence suggests that female sex may be associated with a significant lower risk of in-hospital mortality (37%, p < 0.001), fewer cardiopulmonary and venous thromboembolic complications (27-45% based on complication type, p < 0.021) and surgical site infection (22%, p < 0.032) following TSCI (Mohammad et al., 2024); yet the available evidence remains inconsistent, with studies in literature reporting both worse and better outcomes in females (Benedict et al., 2025). Preclinical studies suggest sex differences at both cellular recruitment level and gene expression in the inflammatory response after TSCI (Stewart et al., 2020, 2021; Ghosh et al., 2024), reflecting a neuroprotective and immune-modulating effect possibly related to sex hormones (Li et al., 2022; Osimanjiang et al., 2022; Stewart et al., 2020). Important to note, sex differences in mortality are not limited to TSCI; in critical care, females often exhibit lower mortality than males in severe conditions such as shock and sepsis (Rose et al., 2025; Bosch et al., 2018).

Traumatic injury is highly heterogeneous, and biological and clinical factors, such as neurological level of injury, injury mechanism, and injury severity, are likely to contribute to outcomes in TSCI patients. However, up until now, a thorough synthesis of mortality data encompassing all TSCI types has been missing. To address this uncertainty, we conducted a systematic review and meta-analysis to evaluate the relationship between sex and mortality after TSCI.

## 2 Materials and methods

### 2.1 Protocol and registration

We conducted a systematic review and meta-analysis according to PRISMA (Liberati et al., 2009; Moher et al., 2009; Page et al., 2021) and MOOSE standards (Stroup et al., 2000). All steps, were pre-defined in the study protocol registered at the International Prospective Register of Systematic Reviews (PROSPERO) with number CRD42021234582.

### 2.2 Eligibility criteria

Studies were eligible for inclusion if sex-based differences in mortality after traumatic injury in humans were evaluated allowing sufficient data to calculate effect sizes. Exclusion criteria included non-English publications, non-full-text articles, studies on burns, poisoning, snake bites, not meeting study type criteria (meta-analysis, review, editorial, experimental study, discussion, letter, case report, or conference abstract), or studies employing specific interventions altering mortality rates.

### 2.3 Information sources and search strategy

Databases PubMed, Embase, Web of Science Core Collection, and Cochrane Library were systematically searched up until June 4, 2025 in collaboration with a medical information specialist (LS). No methodological filters or date restrictions were applied. Search terms combined index terms and free-text words, covering synonyms and related concepts for traumatic injury and sex-related differences in mortality. The full search can be found in Appendix A. Duplicates were removed via Endnote (René et al., 2019; Bramer et al., 2016). Reference lists and Google Scholar were searched for additional relevant literature.

Initially, the search was aimed at the overall trauma population; however, before conducting any formal analyses, the focus was narrowed to TSCI due to the abundance of studies and the wide-ranging nature of trauma. Since TSCI is a distinct clinical condition (Barbiellini Amidei et al., 2022), merging data with other traumatic injuries would reduce the clinical relevance of the findings.

### 2.4 Study selection

Three reviewers (EB, RD, FM) independently screened titles and abstracts. Full texts were assessed by two reviewers (EB, FM), with conflicts resolved through discussion or, if needed, by consulting a third reviewer (PS).

### 2.5 Data extraction

Study characteristics and outcome data were collected by one reviewer (EB) using a pre-piloted standardized form and verified by a second reviewer (FM). Data extraction included the following: 1) study details (author, year, country, design, study period, in-/exclusion criteria, population, and sex-specific sample sizes), 2) patient and injury details (age, injury type, severity, and mechanism, neurological level of injury), and 3) mortality outcomes (crude, adjusted when available, and corresponding confidence intervals and p-values). To resolve any discrepancies, a third reviewer (PS) was consulted.

### 2.6 Assessment of study quality and bias within studies

Methodological quality was independently scored by two reviewers (EB, FM), with a third (PS) resolving disagreements. Study quality was assessed using a modified version of the Newcastle-Ottawa Scale (NOS). Following prior methodological work (Wells et al., 2000; Liu et al., 2015), two additional domains, population size and study design, were incorporated. The comparability domain was specifically adapted to reflect our objective of estimating the total association between sex and mortality following TSCI. Variables such as injury severity and mechanism may function as downstream mediators in this association; consequently, statistical adjustment for these factors could lead to underestimation of the true sex-related effect (Schober and Vetter, 2020a). Moreover, adjusted effect estimates were inconsistently reported and based on heterogeneous covariate sets across studies. Therefore, studies were not scored on the basis of statistical adjustment but were instead evaluated on the transparency of sex-stratified baseline reporting. The adapted NOS is detailed in Appendix B.

### 2.7 Data synthesis and statistical analysis

The primary measure was the odds ratio (OR) for mortality in female versus male TSCI patients. Pooled analyses were conducted using random effects models in STATA 19.0 (StataCorp, College Station, Texas), accounting for anticipated between-study heterogeneity (DerSimonian and Laird, 1986). Summary estimates and corresponding 95% confidence intervals were calculated using crude mortality data. With overlapping populations, only the analysis yielding the highest precision, defined by the smallest standard error, was included to prevent duplicate patient representation.

All reported TSCI types and mortality endpoints were combined to obtain an overall pooled estimate. Additional leave-one-out analyses were conducted to determine whether individual studies exerted undue influence on the pooled effect. Statistical significance was defined as a p-value <0.05. Between-study heterogeneity was expressed using the I^2^ statistic (Higgins and Thompson, 2002; Schober et al., 2021), while 95% prediction intervals were calculated to describe the expected range of effects across future studies (Schober and Vetter, 2020b). Potential small-study effects were evaluated by constructing funnel plots of the log-transformed odds ratios against their standard errors, supplemented by formal testing for asymmetry using Egger's regression (Egger et al., 1997).

## 3 Results

### 3.1 Study selection

**Fig. 1:**
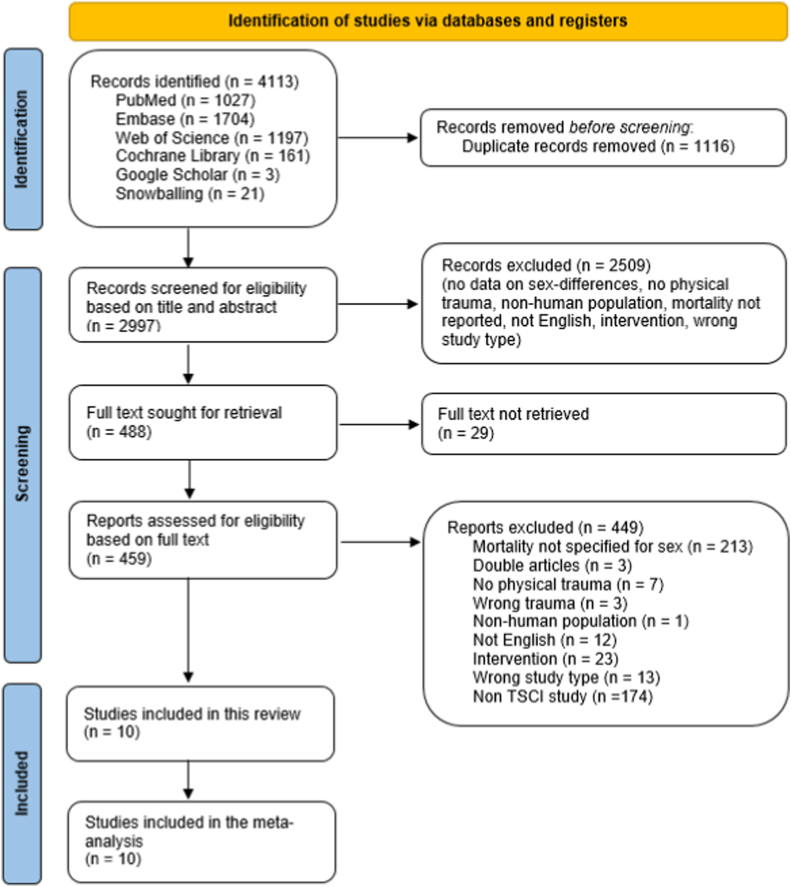
PRISMA flow diagram. PRISMA flow diagram summarizing identification, screening, eligibility and inclusion of studies.

In total, 4113 records were identified. After title and abstract screening and removing duplicates, 459 records remained for full-text screening. Of these, 10 studies met the inclusion criteria and were consequently included in the systematic review and meta-analysis. The selection process is shown in Fig. 1.

### 3.2 Study characteristics

Ten included studies were published between 2005 and 2024 and encompassed data from almost sixty-four thousand patients (18,788 females and 45,204 males) (Mohammad et al., 2024; Barbiellini Amidei et al., 2022; Cao et al., 2013; Kobayashi et al., 2023; Lenehan et al., 2012; O'Connor, 2005; Shibahashi et al., 2019; Varma et al., 2010; Krause and Saunders, 2010; Sabre et al., 2013). Half of the studies were conducted in the United States of America (n = 5), with the remainder from Europe (n = 2), Oceania (n = 1) and Asia (n = 2). All included studies were retrospective cohort studies (Table 1). The NOS rating ranged between six and nine stars, with a median of seven (Table 2).

**Table 1:** General study characteristics.

First Author	Year of publication	Country	Study design	Study in-/exclusion criteria	Study population	N female/male
Barbiellini Amidei	2022	Italy	Retrospective population-based study	1Jan2011-31Dec2020	TSCI	413/890
				All patients at the first hospital admission for primary or secondary diagnosis of spinal cord injury with vertebral fracture (ICD-9- CM 806.x) or without evidence of vertebral fracture (ICD-9-CM 952.x) admitted to any hospital in the Region, alive at time of hospital admission, resident in Vento Region and insured by the regional health service, and only TSCI hospitalizations that followed an ED visit or an urgent admission to a neurosurgery department were included		
				Exclusion: hospitalized patients with TSCI that were not present in the population registry, cases with a diagnosis of spinal column fracture without spinal cord injury, and patients with conditions indicative of a non-TSCI (pathologic fracture of vertebrae (ICD-9-CM 733.13) or secondary malignant neoplasm of the bone and bone marrow (ICD-9-CM 198.5). diagnoses retrieved from planned hospitalizations or admissions to rehabilitation facilities		
Cao	2013	USA	Retrospective cohort study (SCISR, 62 Centers)	1Jan1998-31Dec2009	TSCI	690/1995
				All patients with acute TSCI who were discharged alive		
Ismail	2024	USA	Retrospective cohort study	2013-2019	TS(C)I (isolated, blunt)	14,017/29,739
				Adult (≥18years) patients who suffered an isolated spine injury (Spine AIS≥2 and an AIS≤1 in the remaining regions) as result of a blunt trauma and were managed surgically		
				Exclusion: sex not recorded in the dataset, Spine AIS = 6 (lethal)		
Kobayashi	2023	Japan	Retrospective cohort study (78 Centers)	2010-2020	TSCI (cervical)	505/1007 (overall) 272/631 (underwent surgery)
				Geriatric (≥65years) patients who received inpatient treatment for traumatic cervical spine injuries ((1) isolated cervical fractures and/or dislocations, (2) isolated spinal cord injury, and (3) spinal cord injury with fractures and/or dislocations) and were followed for at least 3 months		
				Exclusion: patients whose treatment course and outcome were unclear		
Krause	2010	USA	Retrospective study (Single Center)	Jul1997-Apr1998	TSCI	339/964
				Adult (≥18years) patients with TSCI, residual deficits, and 1 year post onset. Mortality status was determined as of December 31, 2007, using the National Death Index.		
				Exclusion: questionable diagnosis or missing date of injury or age		
Lenehan	2012	Canada	Retrospective observational study utilizing prospectively collected population-based data (Single Level 1 Trauma Center)	Jan1995-Dec2004	TSCI	186/744
				All patients admitted with TSCI (ICD-9: 952.x and 806.x)		
				Exclusion: patients with neurological deficits secondary to nontraumatic conditions such as tumors, infections, vascular abnormalities, and psychogenic paralysis		
O'Connor	2005	Australia	Retrospective population-based study (6 hospitals)	1Jan1986-31Dec1997	TSCI	577/2315
				Adult (>15years) patients with TSCI, resident and sustained TSCI in Australia		
				Exclusion: iatrogenic and comiogenic cause of TSCI		
Sabre	2012	Norway and Estonia	Retrospective population-based study (8 Norwegian and 22 Estonian hospitals)	1997-2001	TSCI	51/264 (overall) 16/55 (Norway) 35/209 (Estonia)
				All patients admitted with ICD-10 suggesting a TSCI or a fracture of the spinal column at discharge, caused by: motor vehicle accidents, falls, sport injuries and other injuries. All the patients were followed until death or 14 October 2011.		
Shibahashi	2019	Japan	Retrospective cohort study (JTDB, 260 Centers)	2004-2015	TSCI (cervical, thoracic, and/or lumbar)	1509/5794
				Adult (≥18years) patients who were identified with AIS codes indicating cervical spinal cord injury (cervical spinal cord contusion and/or laceration, thoracic spinal cord contusion and/or laceration, lumbar spinal cord contusion and/or laceration, cauda equina contusion and/or laceration)		
				Exclusion: patients who were in cardiopulmonary arrest (SBP = 0 mmHg) on arrival at the hospital		
Varma	2010	USA	Retrospective cohort study	1993-2003	TSCI	501/1493
				All patients with TSCI		

AIS: Abbreviated Injury Scale, ED: Emergency Department, ICD: International Classification of Diseases, SBP: Systolic Blood Pressure, SCISR: Spinal Cord Injury Surveillance Registry, TSCI: Traumatic Spinal Cord Injury.

**Table 2:** Quality assessment.

		Se	C	O	Si	St	☆	Quality
Barbiellini Amidei	2022	4	0	3	0	0	7	Poor
Cao	2013	4	0	2	0	0	6	Poor
Ismail	2024	4	1	3	1	0	9	Good
Kobayashi	2023	4	0	3	0	0	7	Poor
Krause	2010	4	0	3	0	0	7	Poor
Lenehan	2012	4	0	2	0	0	6	Poor
O'Connor	2005	4	0	3	0	0	7	Poor
Sabre	2012	4	0	3	0	0	7	Poor
Shibahashi	2019	4	0	2	1	0	7	Poor
Varma	2010	4	0	3	0	0	7	Poor

Se: Selection (max. 4☆), C: Comparability (max. 2☆), O: Outcome (max. 3☆), Si: Size (max. 1☆), St: Study design (max. 1☆). Because study quality was assessed using categorical ratings, studies with the same number of allocated stars could still differ in overall quality classification (poor, fair, or good).

### 3.3 Patient and injury characteristics: age and type, severity and mechanism of injury

Males dominated the study population (Table 1). Age distribution varied across studies; four studies focused on adults (Mohammad et al., 2024; O'Connor, 2005; Shibahashi et al., 2019; Krause and Saunders, 2010). one on older patients (≥65 years) (Kobayashi et al., 2023), while the others applied no age restrictions (Barbiellini Amidei et al., 2022; Cao et al., 2013; Lenehan et al., 2012; Varma et al., 2010; Sabre et al., 2013). In studies that reported age separately for sex, females were generally older than their male counterparts (Supplemental Table 1). Different TSCI types were studied; acute TSCI (n = 1), isolated spine injury (spine AIS>2) (n = 1), traumatic cervical spine injury (n = 1) and cervical-thoracic and/or lumbar TSCI (n = 1), while six studies did not specify TSCI type. Two studies reported the Injury Severity Score (ISS, Supplemental Table 1): over half of the patients had an ISS greater than 15 in one study, and the other study reported a median ISS of 9. In both studies, females had lower ISS scores compared to males. One study reported that solely blunt trauma injury mechanisms were included, while the remaining studies did not provide data on injury mechanisms.

### 3.4 Summary of results from individual studies: mortality

Mortality was the primary outcome in all ten included studies (Table 3). The time of mortality endpoints varied, with almost half of the studies reporting in-hospital mortality (Mohammad et al., 2024; Lenehan et al., 2012; Shibahashi et al., 2019; Varma et al., 2010), while the remainder assessed post-discharge mortality (mortality at discharge (Cao et al., 2013), at 90 days (Kobayashi et al., 2023), at 1 year (O'Connor, 2005), at 10-years (Krause and Saunders, 2010; Sabre et al., 2013) and one study reported mortality at both 6 months and 1 year (Barbiellini Amidei et al., 2022)).

**Table 3:** Mortality (organized as female/male).

First Author	Year of publication	Mortality Primary outcome?	Mortality percentage	Mortality (un)adjusted variables	P-value	Mortality OR/HR/RR	Significance	Conclusion mortality
Barbiellini Amidei	2022	Yes: 1-month, 6-month, 1-year	17.7%/17.0%	**Crude** 1-year mortality	P = 0.7522	X	ns	ND
			X	**Adjusted** 1-year mortality For age, cause, setting, hospital management, level of the injury, year of event	P = ns	♂HR 1.23 [95% CI 0.91-1.66]	ns	ND
			5.8%/6.2%	**Crude** 1-month mortality	P = ns	X	ns	ND
			X	**Adjusted** 1-month mortality For age, cause, setting, hospital management, level of the injury, year of event	P = ns	♂HR 1.08 [95% CI 0.65-1.78]	ns	ND
			14.8%/13.8%	**Crude** 6-month mortality	P = ns	X	ns	ND
			X	**Adjusted** 6-month mortality For age, cause, setting, hospital management, level of the injury, year of event	P = ns	♂HR 1.14 [95% CI 0.82-1.58] (No ORs reported)	ns	ND
Cao	2013	Yes: after discharge (4288day)	19.9%/15.0%	**Crude** after discharge mortality (4288day)	P = sig	X	**sig**	**Females ↑ Mortality**
			X	**Adjusted** after discharge mortality (4288day) For age	P = 0.03	♂HR 1.3 [95% CI 1.0-1.6] (No ORs reported)	**sig**	**Females ↓ Mortality**
Ismail	2024	Yes: in-hospital	1.5%/2.5%	**Crude** in-hospital mortality	P < 0.001	X	**sig**	**Females ↓ Mortality**
			X	**Adjusted** in-hospital mortality For: All variables were balanced after IPW, with ASDs<1.0. Weights are based on age, race, highest AIS score in each region, level of injury, presence of spinal cord injury, level of spine surgery, hypertension, previous myocardial infarction, congestive heart failure, history of peripheral vascular disease, cerebrovascular disease, dementia, COPD, smoking status, chronic renal failure, diabetes mellitus, cirrhosis, coagulopathy, currently receiving chemotherapy for cancer, metastatic cancer, drug use disorder, alcohol use disorder, major psychiatric illness, and advanced directives limiting care	P < 0.001	♀RR 0.63 [95% CI 0.57-0.69] (No ORs reported)	**sig**	**Females ↓ Mortality**
Kobayashi	2023	Yes: 90-day	1.8%/5.2%	**Crude** 90-day mortality	P = 0.001	X	**sig**	**Females ↓ Mortality**
			X	**Adjusted** 90-day mortality For age at injury, cervical spine fracture, DISH, AIS at admission, respiratory disease, cardiac disease, chronic kidney disease, surgical treatment	P = 0.009	♂OR 3.7 [95% CI 1.5-9.3]	**sig**	**Females ↓ Mortality**
			1.5%/3.2%	**Crude** 90-day mortality **underwent surgery**	P = 0.18	X	ns	ND
Krause	2010	Yes: 10-year	19.5%/21.8%	**Crude** 10-year mortality	P = 0.3868	♂OR 1.15 [95% CI 0.84-1.56]	ns	ND
Lenehan	2012	Yes: in-hospital	4.3%/2.8%	**Crude** in-hospital mortality	P = ns	X	ns	ND
O'Connor	2005	Yes: 1-year	11.1%/11.8% X	**Crude** 1-year mortality	P = ns	♂HR 1.07 [95% CI 0.82-1.41]	ns	ND
				**Adjusted** 1-year mortality For age at injury, cause of injury, neurologic level, extent (complete vs incomplete)	P = sig	♂HR 1.40 [95% CI 1.06-1.85] (No ORs reported)	**sig**	**Females ↓ Mortality**
Sabre	2012	Yes: 10-year	35.3%/35.6% 37.5%/36.4% 34.3%/35.4%	**Crude** 10-year mortality	P = ns	X	ns	ND
				**Crude** 10-year mortality **Norway**	P = ns	X	ns	ND
				**Crude** 10-year mortality **Estonia**	P = ns	X	ns	ND
				**Adjusted** 10-year mortality **Norway** For age at injury, etiology of injury, neurological level, completeness, anatomical level, traumatic brain injury, alcohol consumption	P = 0.89	♂HR 0.94 [95% CI 0.38-2.34]	ns	ND
				**Adjusted** 10-year mortality **Estonia** For age at injury, etiology of injury, neurological level, completeness, anatomical level, traumatic brain injury, alcohol consumption	P = 0.99	♂HR 1.00 [95% CI 0.55-1.85] (No ORs reported)	ns	ND
Shibahashi	2019	Yes: in-hospital	4.1%/6.1%	**Crude** in-hospital mortality	P = sig	X	**sig**	**Females ↓ Mortality**
			X	**Adjusted** in-hospital mortality For year of admittance, age, etiology, GCS score on arrival, hypotension on arrival, bradycardia on arrival, severe head injury, ISS, level of spinal cord injury, neurological severity of spinal cord injury	P < 0.001	♂OR 2.06 [95% CI 1.44-2.93]	**sig**	**Females ↓ Mortality**
Varma	2010	Yes: in-hospital	11.4%/13.0%	**Crude** in-hospital mortality	P = 0.35	♂OR 1.2 [95% CI 0.9-1.6]	ns	ND
			X	**Adjusted** in-hospital mortality For race, age, Frankel grade, comorbidities, injury level, ISS category, TBI, trauma center level	P = 0.016	♂OR 1.6 [95% CI 1.1-2.2]	**sig**	**Females ↓ Mortality**

ND: No Difference, NR: Not Reported, ns: not statistically significant.

### 3.5 Overall mortality: crude and adjusted

Of the ten studies (Table 3), three (Mohammad et al., 2024; Kobayashi et al., 2023; Shibahashi et al., 2019) reported lower crude mortality for females compared to males, and this association persisted after adjusting for covariates. Six studies (Barbiellini Amidei et al., 2022; Lenehan et al., 2012; O'Connor, 2005; Varma et al., 2010; Krause and Saunders, 2010; Sabre et al., 2013) found no difference in crude mortality. After adjustment, two studies reported no significant difference in mortality (Barbiellini Amidei et al., 2022; Sabre et al., 2013), whereas two studies found lower mortality among females (O'Connor, 2005; Varma et al., 2010). Two studies did not perform an adjusted analysis (Lenehan et al., 2012; Krause and Saunders, 2010). One study (Cao et al., 2013) initially reported higher crude mortality in females; nevertheless, after adjustment, this effect reversed, with females showing a lower mortality rate than males (Supplemental Fig. 1).

### 3.6 Subgroup: type and level of spinal cord injury and mechanism of injury

One study (Kobayashi et al., 2023) specifically focused on cervical spinal cord injury, whereas Shibahashi et al. stratified patients by cervical, thoracic, and lumbar TSCI; however, mortality by sex was only reported for the overall group. Ismail et al. examined patients with isolated spinal cord injury and found lower mortality in females compared to males, while Cao et al. investigated acute TSCI, reporting conflicting outcomes. The remaining six studies did not specify the type of TSCI. Ismail et al., specifically included patients with blunt trauma and found lower mortality in females compared to males (Mohammad et al., 2024). The other studies did not specify what trauma mechanism was included. ISS and AIS based subgroup analyses were not performed in included studies.

### 3.7 Pooled results: meta-analysis and sensitivity analyses

**Fig. 2:**
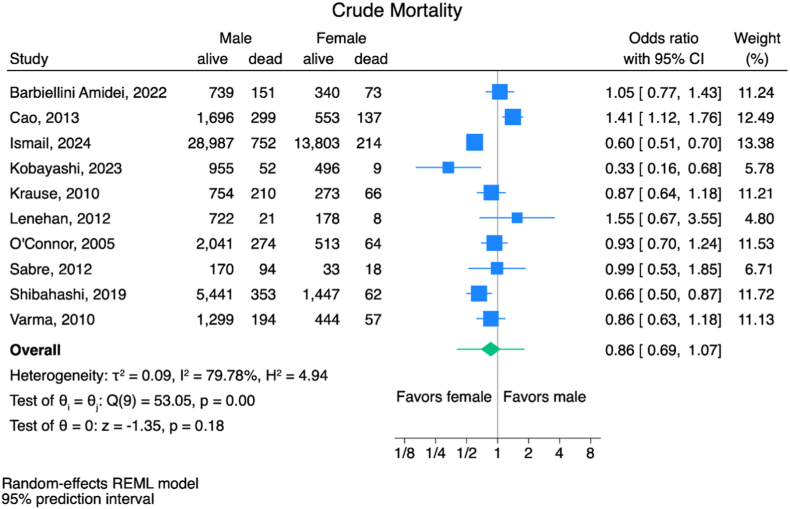
Forest plot. Forest plot summarizing the individual studies and pooled results of the meta-analysis.

All ten studies were included in the meta-analysis (Table 1). Substantial heterogeneity was observed between all studies (I^2^ 79.78%, t^2^ 0.09). Egger's test showed no evidence for small study bias (p = 0.942, Supplemental Fig. 2). No overall significant association between sex and TSCI mortality was found (OR 0.86, 95% CI 0.69-1.07, 95% PI 0.41-1.80, p = 0.18, Fig. 2). To assess the robustness of our findings, a leave-one-out sensitivity analysis was performed. This analysis showed that the non-significant result was driven by the study of Cao et al., as the pooled odds ratios for mortality became statistical significant in favor of females after leaving this study out. A sensitivity analysis restricted to type of mortality (in-hospital vs. post-discharge) demonstrated lower mortality among females in studies assessing in-hospital mortality (OR 0.73, 95% CI 0.57-0.93, I^2^ 62.54%, 95% PI 0.27-1.93, t^2^ 0.04, p = 0.01, Supplemental Fig. 3), whereas studies evaluating post-discharge mortality showed no significant sex-related differences (OR 0.93, 95% CI 0.69-1.25, I^2^ 76.29%, 95% PI 0.36-2.42, t^2^ 0.10, p = 0.64, Supplemental Fig. 3).

## 4 Discussion

Sex-related differences in mortality after TSCI were explored by conducting a systematic review and meta-analysis. At the individual study level, six of ten studies reported lower adjusted mortality in females compared to their male counterparts. The overall pooled analyses showed no sex-related differences in mortality following TSCI, however, this result should not be interpreted as evidence that sex has no association with mortality. A non-significant pooled estimate, particularly in the presence of substantial between-study heterogeneity, may reflect variability in direction and magnitude of the association across study contexts rather than a true null effect. The observed heterogeneity (I^2^ 79.78%, t^2^ 0.09) and wide prediction interval (0.41-1.80) further underscore this variability, suggesting that the true effect of sex on mortality may range from a meaningful female survival advantage, through no difference, to a male survival advantage depending on the study context. Indeed, the leave-one-out sensitivity analysis indicated a potential survival advantage for females after exclusion of a single study, and studies reporting in-hospital mortality suggested slightly lower mortality for females. Under a random-effects model, the pooled result reflects an average of heterogeneous effects, and the true relationship between sex and mortality after TSCI likely varies depending on setting, patient selection, injury characteristics, and analytic approach. Specific subgroup analyses, including age categories, injury severity and mechanism, and study quality, could not be performed in this study due to limited and inconsistently reported data. Epidemiological studies demonstrate clear sex differences in TSCI incidence with females affected at approximately 13 cases per million people, whereas the incidence in males is roughly three times higher, at about 42 per million (Lu et al., 2024). This marked male predominance in TSCI research has shaped therapeutic approaches largely for males. While this reflects the higher incidence of TSCI among males, the growing emphasis on personalized medicine highlights the need to consider potential sex-specific differences in treatment and recovery, as females may receive less tailored care. In particular, sex-related differences reported in the literature, such as those in neuroinflammatory responses and functional recovery following TSCI, may indicate opportunities to further refine therapeutic strategies.

The observed trend toward lower in-hospital mortality in females is clinically relevant and may reflect several underlying mechanisms. Estrogen-mediated neuroprotection and immune modulation may attenuate secondary injury in the acute phase, and females may exhibit a more favorable cardiopulmonary response to trauma (Stewart et al., 2020, 2021; Li et al., 2022; Osimanjiang et al., 2022; Datto et al., 2015; Benedict et al., 2026; Mohammad et al., 2024; Ghosh et al., 2024; Bosch et al., 2018). These sex-specific cardiopulmonary differences may be particularly relevant at higher injury levels, where autonomic dysfunction and respiratory compromise are known to substantially contribute to mortality risk. These biological mechanisms are likely most consequential during the acute and subacute phases of injury, which may explain why a sex-based survival advantage appears more consistently in in-hospital settings than in long-term follow-up. It should be noted, nevertheless, that these hormonal effects may be substantially attenuated in postmenopausal females, in whom circulating estrogen levels are considerably reduced, potentially limiting the neuroprotective and immune-modulatory benefits observed in premenopausal female patients. In longer-term outcomes, competing factors such as frailty, comorbidities, socioeconomic status, access to rehabilitation and healthcare utilization likely play an increasingly dominant role in determining survival, potentially obscuring any sex-based biological advantage. Additionally, survivor bias cannot be excluded as a contributing factor to the attenuation of sex-based differences in longer-term outcomes, as a differential acute mortality between sexes may result in systematically different survivor populations across follow-up studies.

Although not primarily focusing on sex differences, two previous studies looked into mortality after TSCI in a systematic review, taking into account sex as a factor. Chamberlain et al. reported that males demonstrated higher mortality compared to females after TSCI in pooled analyses of four studies (Chamberlain et al., 2015). Similarly, Zadra et al. evaluated life expectancy after TSCI, with attention to sex (Zadra et al., 2024). Although inclusion criteria required sex-stratified mortality data, the results section provided only a brief narrative summary, based on the same four previously mentioned studies. While their quantitative synthesis provides valuable insights, the current study incorporated the same studies along with six additional ones, resulting in a broader and more representative analysis of the current evidence base on prognosis after TSCI. To our knowledge, the present study provides one of the most comprehensive evaluations to date of sex differences in TSCI-related mortality, synthesizing data from ten studies comprising approximately sixty-thousand patients. By incorporating demographic and injury-related variables to the extent feasible, we aimed to offer a structured and quantitative evaluation of the available evidence.

An important methodological issue in sex-based research concerns how studies accounted for variables associated with both sex and mortality; specifically, the distinction between crude and adjusted analyses. While some studies reported crude outcomes, others adjusted for variables such as injury severity and mechanism. Critically, these variables may lie on the causal pathway between sex and mortality. For instance, if males sustain more severe injuries partly due to behavioral or biomechanical factors correlated with sex, then adjusting for injury severity may constitute overadjustment, removing variation that is itself a manifestation of the sex-mortality relationship. This would result in an estimate of the conditional association between sex and mortality given injury severity, rather than the total causal effect of sex on mortality. Inconsistent reporting of adjusted associations and varying covariate sets across studies further limit comparability. Therefore, we focused on crude, sex-stratified mortality data to capture the total observed effect. Nonetheless, unadjusted estimates remain susceptible to confounding, especially by age, as female patients tend to be older in the existing literature. Interestingly, even when females are generally older, a characteristic associated with higher mortality, no consistent disadvantage in female mortality is observed, which may further support a protective role of female sex in the acute injury setting. Moreover, in the few studies that did report lower injury severity for females, females continued to show lower mortality rates compared to males even after adjusting for injury severity.

This review has several strengths. This is the first systematic review and meta-analysis dedicated to sex-specific mortality outcomes in TSCI patients. We conducted a thorough search across multiple databases, adhered to established methodological standards, and included a large, diverse sample. We quantified heterogeneity using both the I^2^ statistic and prediction intervals, and assessed the robustness of our findings through leave-one-out analyses, offering a transparent overview of evidence variability.

Still, several limitations must be noted. All included studies were observational, making them susceptible to confounding by unmeasured factors. Variables such as socioeconomic status, access to healthcare, pre-existing comorbidities, and hormonal factors (e.g., contraceptive use or hormone replacement therapy) were infrequently reported and could therefore not be accounted for in the analyses. Also, because the included studies reported patients by biological sex without assessment of gender identity or sociocultural gender-related factors, the present review specifically evaluates sex-related rather than gender-related differences. Furthermore, cause of death was not reported in the included studies. This is a relevant limitation, as patients with TSCI are likely to sustain concomitant injuries, including traumatic brain injury, that carry independent mortality risk. Without data on cause of death, it is not possible to determine whether observed sex differences in mortality reflect the direct consequences of TSCI, associated injuries, or other competing causes. Meta-regression and sensitivity analyses on methodological quality, age, injury severity and injury mechanism had been planned, but could not be performed due to sparsity of data. Given that higher injury severity is associated with greater mortality, and that males are known to sustain more severe injuries on average, this represents a potential source of residual confounding that limits the interpretability of the pooled estimate. Similarly, age was not consistently reported in a manner that allowed adjustment across studies, despite its known influence on mortality outcomes after TSCI. These factors should be systematically reported in future studies to enable more robust sex-stratified analyses. Variability in mortality timing and injury severity definitions was present across studies, and the results of the subgroup analysis on type of mortality should be interpreted with caution, as the test for subgroup differences did not reach statistical significance. Moreover, given the limited number of included studies, the observed heterogeneity, and the methodological limitations of the available evidence, the findings of our study should be considered exploratory rather than definitive. Residual heterogeneity in sensitivity and subgroup analyses suggests that unaccounted differences in patient populations, care practices, or healthcare systems may influence outcomes. While a limitation, this also reflects the complexity of real-world clinical practice. Finally, while Egger's test did not suggest significant publication bias, it cannot be fully excluded.

## 5 Conclusion

In conclusion, our findings show no statistically significant overall association between sex and mortality after TSCI. Nevertheless, this result should be interpreted in the context of substantial between-study heterogeneity and methodological variability across included studies. Sensitivity and subgroup analyses suggest lower mortality in females compared to males, a pattern consistent with the qualitative synthesis. The association between sex and mortality following TSCI therefore appears context-dependent, varying by study setting, patient selection, injury characteristics, and analytic choices. Consequently, future studies should investigate the timing, sources, and conditions under which sex influences outcomes in TSCI patients, ideally incorporating standardized definitions of mortality, injury severity, and follow-up duration, with the aim of developing more individualized risk stratification and, where appropriate, sex-specific therapeutic management strategies.

## Data sharing statement

All data analyzed in this systematic review and meta-analysis, including extracted study-level data on patient characteristics, mortality rates, and subgroup variables, are available from the corresponding author upon reasonable request. This includes the data extraction sheets, coding for subgroup analyses, and any statistical code used for the meta-analyses. Access may be provided for academic and research purposes, and requests will be considered on a case-by-case basis to ensure appropriate use.

## Declaration of competing interest

The authors declare that they have no known competing financial interests or personal relationships that could have appeared to influence the work reported in this paper.

## Device Status/Drug Statement

The Manuscript submitted does not contain information about medical device(s)/drug(s).
