# Supplementary material for: Sex differences in patient mortality after traumatic spinal cord injury: A systematic review and meta-analysis
Source: Brain Spine. 2026 Jul 16;6:106176. doi: 10.1016/j.bas.2026.106176 (PMC13418270; doi:10.1016/j.bas.2026.106176)
Supplement: Multimedia component 1 [file mmc1.docx]

**Supplemental Material: Figures and Tables**

**Supplemental Figure 1. Mortality flow diagram.**

Flow diagram summarizing crude and adjusted results on mortality of all included studies.

**Crude analysis (n=10 studies)**

**Females ↓ Mortality**

N=3

**No differences**

N=6

**Females ↑ Mortality**

N=1

**After adjusted analysis**


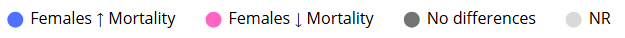


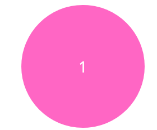

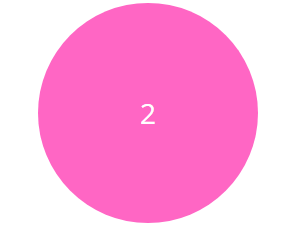

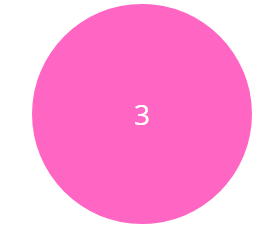


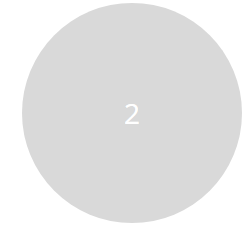


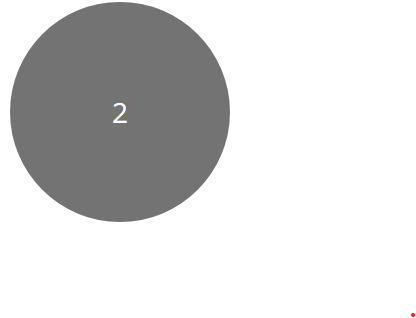


NR: not reported.

**Supplemental Figure 2. Funnel plot.**

Funnel plot of included studies.


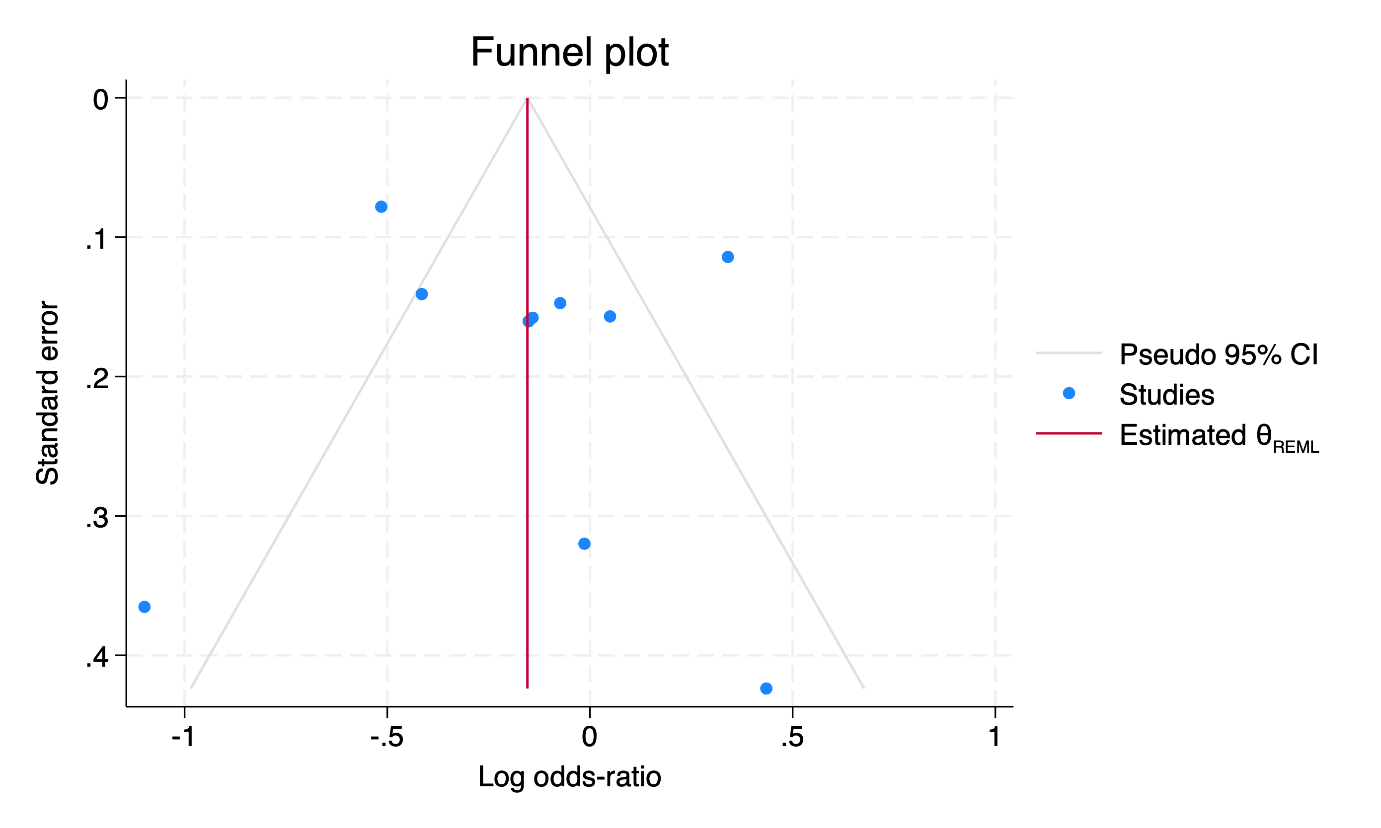


**Figure 3. Forest plot, stratified on mortality type.**

Forest plot summarizing the individual studies and pooled results of the meta-analysis, stratified for mortality type.


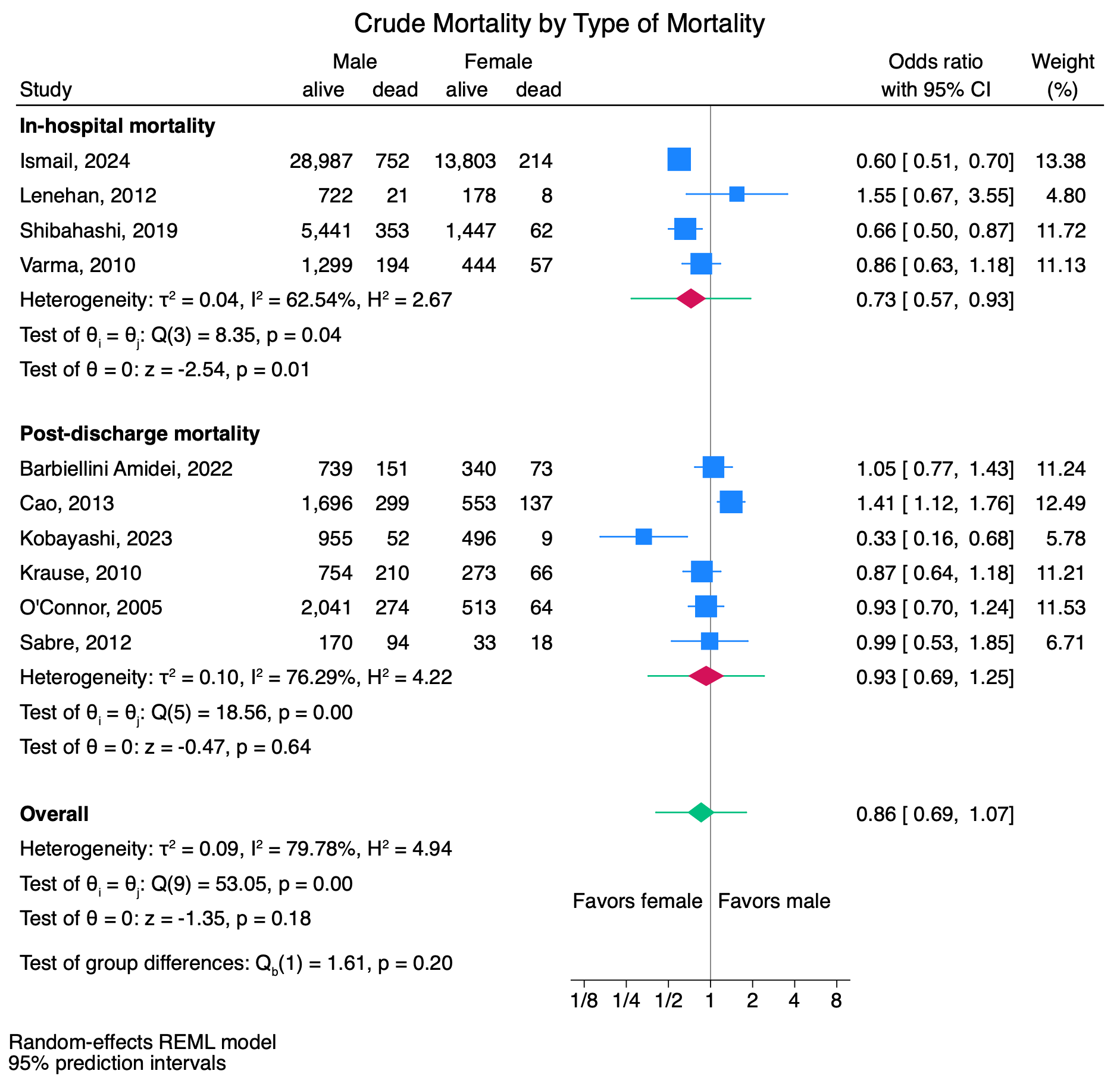


**Supplemental Table 1**. **Age, ISS and mechanism (organized as female/male)**

| First Author | Year of publication | Age in years (mean±SD or median(IQR)) | P-value | ISS (mean±SD or median(IQR)) and/or other scores for injury severity | P-value | Mechanism (blunt or penetrating) | *P-value* | *Conclusion* |
| --- | --- | --- | --- | --- | --- | --- | --- | --- |
| Barbiellini Amidei | 2022 | NR (overall cohort: 59.2±21.4) | P=NR | NR | P=NR | NR | P=NR | NR |
| Cao | 2013 | 50.2/42.8 | P=sig | NR | P=NR | NR | P=NR | Females ↑age |
| Ismail | 2024 | 59(41-73)/55(38-68) | P<0.001 | 9.0(5.0-13)/9.0(5.0-16) | P<0.001 | N/A (all blunt) | N/A (all blunt) | Females ↑age ↓ISS |
| Kobayashi | 2023 | NR (overall cohort: 75.8±6.9) | P=NR | NR | P=NR | NR | P=NR | NR |
| Krause | 2010 | NR (overall cohort: 31.7(13.8)) | P=NR | NR | P=NR | NR | P=NR | NR |
| Lenehan | 2012 | NR (overall cohort 26.9±13.9) | P=NR | NR | P=NR | NR | P=NR | NR |
| O’Connor | 2005 | NR (most subjected were <45y) | P=NR | NR | P=NR | NR | P=NR | NR |
| Sabre | 2012 | NR (overall cohort Norway: 48.9±23, overall cohort Estonia: 38.9±17.3) | P=NR | NR | P=NR | NR | P=NR | NR |
| Shibahashi | 2019 | NR (overall cohort: 63(49-73)) | P=NR | NR | P=NR | NR | P=NR | NR |
| Varma | 2010 | NR (overall cohort 41(27-57)) | P=NR | *ISS>15:* 41%/50% | P=NR | NR | P=NR | *ISS>15*: Females ↓ISS |

ISS: Injury Severity Score, NR: Not Reported

**Appendix A**

| **Search** | **PubMed Query – June 4, 2025** | **Results** |
| --- | --- | --- |
| #1 | "Sex Factors"[Mesh] OR "Sex Characteristics"[Mesh] OR "Sex Distribution"[Mesh] OR "sex inequalit*"[tiab] OR "sex bias"[tiab] OR "sex diff*"[tiab] OR "sex dimorph*"[tiab] OR "sex-specific*"[tiab] OR "sex based"[tiab] OR "sex disparit*"[tiab] OR "sex factor*"[tiab] OR "sex characteristic*"[tiab] OR "sex dichotom*"[tiab] OR "sex related"[tiab] OR "gender inequalit*"[tiab] OR "gender bias"[tiab] OR "gender diff*"[tiab] OR "gender dimorph*"[tiab] OR "gender specific*"[tiab] OR "gender based"[tiab] OR "gender disparit*"[tiab] OR "gender factor*"[tiab] OR "gender characteristic*"[tiab] OR "gender dichotom*"[tiab] OR "gender related"[tiab] OR "male versus female"[tiab] OR "men versus women"[tiab] OR "men and women"[tiab] OR "women and men"[tiab] OR "male and female"[tiab] OR "female and male"[tiab] | 636,311 |
| #2 | "Shock, Hemorrhagic"[Mesh] OR "Hypovolemia"[Mesh] OR "Trauma Centers"[Mesh] OR "trauma center*"[tiab] OR "trauma centre*"[tiab] OR "trauma regist*"[tiab] OR "hypovolem*"[tiab] OR "severe trauma*"[tiab] OR "severely injured"[tiab] OR "massive transfusion"[tiab] OR (("hemorrhag*"[tiab] OR "haemorrhag*"[tiab] OR "bleeding*"[tiab] OR "blood loss"[tiab]) AND ("shock"[tiab] OR "trauma"[tiab] OR traumatic*[tiab] OR "incident"[tiab] OR "injur*"[tiab] OR "accident"[tiab])) | 154,225 |
| #3 | "Mortality"[Mesh] OR "Hemorrhage/mortality"[Mesh] OR "Wounds and Injuries/mortality"[Mesh] OR "mortalit*"[tiab] OR "survival outcome"[tiab] OR "survival rate*"[tiab] OR "death rate*"[tiab] OR "case fatality rate*"[tiab] | 1,574,364 |
| #4 | #1 AND #2 AND #3 | 1,060 |
| #5 | #4 NOT ("Animals"[Mesh] NOT "Humans"[Mesh]) | 1,027 |

| **Search** | **Embase.com Query – June 4, 2025** | **Results** |
| --- | --- | --- |
| #1 | 'sex ratio'/exp OR 'sex difference'/exp OR ((('sex' OR 'gender') NEAR/3 ('inequalit*' OR 'bias' OR 'diff*' OR 'dimorph*' OR 'specific*' OR 'based' OR 'disparit*' OR 'factor*' OR 'characteristic*' OR 'dichotom*' OR 'related')):ti,ab,kw) OR ((('male' OR 'males' OR 'men' OR 'man') NEAR/3 ('female' OR 'females' OR 'woman' OR 'women')):ti,ab,kw) | 1,609,749 |
| #2 | 'hemorrhagic shock'/exp OR 'hypovolemia'/exp OR 'hospital emergency service'/exp OR 'hypovolem*':ti,ab,kw OR 'trauma center*':ti,ab,kw OR 'trauma centre*' OR 'trauma regist*' OR 'severe trauma*':ti,ab,kw OR 'severely injured':ti,ab,kw OR 'massive transfusion':ti,ab,kw OR ((('hemorrhag*' OR 'haemorrhag*' OR 'bleeding' OR 'blood') NEAR/3 ('shock' OR 'trauma' OR 'incident' OR 'injur*' OR 'accident')):ti,ab,kw) | 151,217 |
| #3 | 'mortality'/exp OR 'hospital mortality'/exp OR 'mortality rate'/exp OR 'mortalit*':ti,ab,kw OR 'survival outcome':ti,ab,kw OR 'survival rate*':ti,ab,kw OR 'death rate*':ti,ab,kw OR 'case fatality rate*':ti,ab,kw | 2,476,478 |
| #4 | #1 AND #2 AND #3 | 2,299 |
| #5 | #4 NOT ([animals]/lim NOT [humans]/lim) | 2,261 |
| #6 | #5 NOT ('clinical trial'/it OR 'conference abstract'/it OR 'conference review'/it) | 1,704 |

| **Search** | **Clarivate Analytics/Web of Science Core Collection Query – June 4, 2025  *Indexes=SCI-EXPANDED, SSCI, A&HCI, ESCI Timespan=All years*** | **Results** |
| --- | --- | --- |
| #1 | TS=((("sex" OR "gender") NEAR/3 ("inequalit*" OR "bias" OR "diff*" OR "dimorph*" OR "specific*" OR "based" OR "disparit*" OR "factor*" OR "characteristic*" OR "dichotom*" OR "related") ) OR (("male" OR "males" OR "men" OR "man") NEAR/3 ("female" OR "females" OR "woman" OR "women") )) | 1,194,844 |
| #2 | TS=("hypovolem*" OR "trauma center*" OR "trauma centre*" OR "trauma regist*" OR "severe trauma*" OR "severely injured" OR "massive transfusion" OR (("hemorrhag*" OR "haemorrhag*" OR "bleeding" OR "blood") NEAR/3 ("shock" OR "trauma" OR "incident" OR "injur*" OR "accident"))) | 95,917 |
| #3 | TS= ("mortalit*" OR "survival outcome" OR "survival rate*" OR "death rate*" OR "case fatality rate*") | 1,749,777 |
| #4 | #1 AND #2 AND #3 | 1,197 |

| **Search** | **Wiley/Cochrane Library Query – June 4, 2025** | **Results** |
| --- | --- | --- |
| #1 | ((sex NEXT inequalit*) OR "sex bias" OR (sex NEXT diff*) OR (sex NEXT dimorph*) OR (sex NEXT specific*) OR "sex based" OR (sex NEXT disparit*) OR (sex NEXT factor*) OR (sex NEXT characteristic*) OR (sex NEXT dichotom*) OR "sex related" OR (gender NEXT inequalit*) OR "gender bias" OR (gender NEXT diff*) OR (gender NEXT dimorph*) OR (gender NEXT specific*) OR "gender based" OR (gender NEXT disparit*) OR (gender NEXT factor*) OR (gender NEXT characteristic*) OR (gender NEXT dichotom*) OR "gender related" OR "male versus female" OR "men versus women" OR "men and women" OR "women and men" OR "male and female" OR "female and male") :ti,ab,kw (word variations have been searched) | 51975 |
| #2 | (hypovolem* OR (trauma NEXT center*) OR (trauma NEXT centre*) OR (trauma NEXT regist*) OR (severe NEXT trauma*) OR "severely injured" OR "massive transfusion" OR (("hemorrhag*" OR "haemorrhag*" OR "bleeding" OR "blood loss") AND ("shock" OR "trauma" OR "incident" OR injur* OR "accident"))) :ti,ab,kw (word variations have been searched) | 32450 |
| #3 | (mortalit* OR ("survival" NEXT (outcome OR rate*)) OR (death NEXT rate*) OR (case NEXT fatality NEXT rate*)) :ti,ab,kw (word variations have been searched) | 145796 |
| #4 | #1 AND #2 AND #3 | 161 |

| **Search** | **Google Scholar – Sep 6, 2025** | **Results** |
| --- | --- | --- |
|  | Severe trauma\|severe injury\|hemorrhagic shock+ ”sex\|gender+differences\|dimorphism\|dichotomy\|specific\|characteristics\|factor\|bias\|inequalities\|disparities”+mortality\|survival rate\|death rate | In accordance with the protocol, the first 200 hits were analyzed |

**Appendix B**

| **Assessment of quality of a cohort study – Newcastle Ottawa Scale (NOS)** |
| --- |
| **Selection maximum 4 stars** |
| 1. Representativeness of the female cohort (with respect to the male cohort)  a) truly representative = ☆  b) somewhat representative = ☆  c) selected group = 0  d) no description of the derivation of the cohort = 0 |
| 2. Selection of the female cohort  a) drawn from the same community as the male cohort = ☆  b) drawn from a different source = 0  c) no description of the derivation of the non intervention cohort = 0 |
| 3. Ascertainment of sex (female or male)  a) secure record (eg surgical record) = ☆  b) structured interview = ☆  c) written self report = 0  d) other / no description = 0 |
| 4. Demonstration that mortality was not present at start of study  a) yes = ☆  b) no = 0 |
| **Comparability maximum 2 stars*** |
| 1. Comparability of cohorts on the basis of the design or analysis: studies reported baseline values of females and males regarding to age, injury severity score, mechanism of injury  a) 3 out of 3 = ☆☆  b) 2 out of 3 = ☆  c) 0-1 out of 3 = 0 |
| **Outcome maximum 3 stars** |
| 1. Assessment of mortality  a) independent blind assessment = ☆  b) record linkage = ☆  c) self report = 0  d) other / no description = 0 |
| 2. Was follow up long enough for mortality to occur  a) yes = ☆ (in-hospital mortality: minimal follow up until discharge, 30-day mortality: 30 days etc.)  b) no = 0 |
| 3. Adequacy of follow up of cohorts  a) complete follow up: all subjects accounted for = ☆  b) subjects lost to follow up unlikely to introduce bias: number lost <= 20%, = ☆  or description of those lost suggesting no different from those followed  c) follow up rate < 80% (select an adequate %) and no description of those lost = 0  d) no statement = 0 |
| **Size maximum 1 star*** |
| 1. Size of the cohort  a) >1.000 patients in each group (female and male) = ☆  b) <1.000 patients in each group (female and male) = 0 |
| **Cohort design maximum 1 star*** |
| 1. Design of the study  a) Prospective cohort = ☆  b) Retrospective cohort (trauma registry) = 0 |
| ** edited fields compared to standard NOS* |

| **Thresholds for converting the NOS to AHRQ standards [https://www.ncbi.nlm.nih.gov/books/NBK115843/bin/appe-fm3.pdf]** |
| --- |
| **Good quality** |
| - 3 or 4 stars in selection domain - 1 or 2 stars in comparability domain - 2 or 3 stars in outcome domain - 1 star in the size domain* - 0 or 1 star in the cohort design domain* |
| **Fair quality** |
| - 2 stars in selection domain - 1 star in comparability domain - 2 or 3 stars in outcome domain - 1 star in the size/cohort design domains combined* |
| **Poor quality** |
| - 0 or 1 star in selection domain - 0 stars in comparability domain - 0 or 1 stars in outcome domain - 0 stars in the size/cohort design domains combined |
| ** edited fields compared to standard NOS* |
